# Relationship between sleep disorders and impairments in the language development process in cases of ASD

**DOI:** 10.1192/j.eurpsy.2025.569

**Published:** 2025-08-26

**Authors:** M. Stilpen, D. Cardilli, D. R. Molini-Avejonas

**Affiliations:** 1 REHABILITATION SCIENCES, USP, SÃO PAULO, Brazil

## Abstract

**Introduction:**

Autism Spectrum Disorder is characterized by persistent deficits in communication and social interaction in multiple contexts, including deficits in social reciprocity, in nonverbal communication behaviors used for social interaction and in skills to develop, maintain and understand relationships,in addition to repetitive patterns of activity. Individuals with Autism Spectrum Disorder (ASD) present a wide variety of cognitive profiles, with levels of symptom severity that differ between those with the same diagnosis. In language development, there are individual differences, both in the acquisition process and in speed and quality. Sleep is an important parameter for child development and its relevance has been associated with healthy physical, cognitive and behavioral development, as well as cognitive functions in children and adolescents. Researchers estimate that between 40% and 80% of children with ASD have sleep disorders.

**Objectives:**

To relate sleep disorders frequently present in cases of ASD to delays in language development in children with ASD.

**Methods:**

The research was approved by the USP ethics committee. To investigate sleep disorders, the Cognitive Speech Therapy Protocol aimed at ASD was used, an accessible and understandable instrument for parents or guardians. To identify language disorders, the ADL2 Language Development Assessment was used. 40 parents and/or guardians of children with ASD, aged between 2 and 12 years, participated in the research. A logistic regression analysis was performed to evaluate the relationship between sleep disturbances and language delay in children with ASD. The sample included 32 children, 28 of whom reported sleep disorders and all of whom had language delays

**Results:**

The results showed that children with sleep disorders are 2.3 times more likely to have delays in receptive and/or expressive language compared to those without sleep disorders. This association is statistically significant, indicating that sleep disorders are a relevant risk factor for language delays in children with ASD.

**Conclusions:**

Sleep disorders can impact the development of expressive language in several ways, such as by reducing the time available for language and interaction practices or by direct effects on cognitive and neurological processes. Therefore, sleep quality should be considered an important factor to be addressed to improve both receptive and expressive language in children with ASD. Therefore, sleep quality should be considered an important factor to be addressed to improve both receptive and expressive language in children with ASD.

**Disclosure of Interest:**

None Declared

